# Experimental Investigation on the Mechanical and Dynamic Thermomechanical Properties of Polyether Ether Ketone Based on Fused Deposition Modeling

**DOI:** 10.3390/polym16213007

**Published:** 2024-10-26

**Authors:** Guocheng Liu, Ning Hu, Junjie Huang, Qiyong Tu, Fengxiang Xu

**Affiliations:** 1 Hubei Key Laboratory of Advanced Technology of Automotive Components, Wuhan University of Technology, Wuhan 430070, China; hn3486572278@163.com (N.H.); huangjunjie@ceec.net.cn (J.H.); tuqiy1688@163.com (Q.T.); 2 Hubei Collaborative Innovation Center for Automotive Components Technology, Wuhan University of Technology, Wuhan 430070, China; 3 State Key Laboratory of Materials Processing and Die & Mould Technology, Huazhong University of Science and Technology, Wuhan 430074, China

**Keywords:** polyether ether ketone (PEEK), fused deposition modeling (FDM), mechanical properties, dynamic mechanical analysis (DMA)

## Abstract

In this work, the mechanical and dynamic thermomechanical properties of PEEK based on FDM are experimentally investigated and evaluated comprehensively. The tensile failure mechanism of PEEK prepared by FDM and extrusion modeling (EM) was analyzed by fracture morphology observation. By conducting a differential scanning calorimetry (DSC) test, the crystallinity of PEEK prepared by FDM and EM was measured. The dynamic thermomechanical properties of PEEK were tested and analyzed by dynamic mechanical analysis (DMA). For FDM-prepared PEEK samples, the yield strength and elongation were 98.3 ± 0.49 MPa and 22.86 ± 2.12%, respectively. Compared with the yield strength of PEEK prepared by EM, the yield strength of PEEK prepared by FDM increased by 65.38%. The crystallinity of FDM-prepared and EM-prepared samples was calculated as 34.81% and 31.55%, respectively. Different processing methods resulted in differences in the microscopic morphology and crystallinity of two types of PEEK parts, leading to differences in mechanical properties. The internal micropores generated during the FDM processing of PEEK significantly reduced the elongation. Moreover, according to the DMA results, the glass transition activation energy of PEEK was obtained as ΔE = 685.07 kJ/mol based on the Arrhenius equation. Due to the excellent mechanical properties of PEEK prepared by FDM processing, it is promising for high-performance polymer applications in different fields.

## 1. Introduction

Polyether ether ketone (PEEK) is a semi-crystalline aromatic polymer with excellent physical and chemical properties. It is also one of the most outstanding special engineering plastics with comprehensive performance and the characteristic of light weight [1,2,3]. Specifically, PEEK is the best high-temperature-resistant polymer material in the existing special engineering plastics and has excellent corrosion resistance, which makes it suitable for automobile batteries and fuel management systems [4,5]. Furthermore, it has excellent biocompatibility and mechanical properties similar to human bones [6,7,8,9,10]. Consequently, PEEK can replace conventional materials such as ceramics and metals on many occasions, such as aerospace engineering and biomedical fields [11].

Additive manufacturing is a technique through which 3D complex objects can be created by the deposition of material, layer by layer [12,13,14]. The additive manufacturing methods for engineering thermoplastics mainly include DIW (Direct Ink Writing) [15,16], SLS (Selective Laser Sintering), and FDM (Fused Deposition Modeling) [17,18]. FDM is one of the most widely used layered manufacturing technologies in various industries due to its capability to create complex parts at the lowest cost [19,20,21]. In light of the unique advantages of FDM and the excellent comprehensive performance of PEEK, the convergence of FDM technology and PEEK is an important way to expand the application range of PEEK to meet industrial needs [22,23,24,25,26].

In the process of manufacturing FDM parts, there are many controllable printing parameters, such as printing temperature (including bed temperature, ambient temperature, and nozzle temperature), printing speed, layer thickness, printing infill angle, filling density, etc. These printing parameters will affect the forming quality and mechanical properties of the parts. Therefore, many researchers have conducted experiments to study the influence of printing parameters on the performance of the FDM-prepared parts. The research results of Karimi et al. [27] indicate that increasing the nozzle temperature can improve the mechanical properties of PBAT. The compressive strength and energy absorption performance of the sample with a filling density of 60% are better than those of the sample with a filling density of 40%. SEM images show that, as the nozzle temperature increases, the printing quality greatly improves. Rahmatabadi et al. [28] 3D-printed pure PVC samples using different printing parameters. Among the printing parameters, the raster angle and printing speed had a significant impact on the mechanical properties, while the layer thickness and nozzle diameter had a relatively small impact. The SEM images also show that printing speed greatly affects the quality of the final component. For the FDM processing of PEEK, Deng et al. [23] conducted a four-factor three-level orthogonal experiment to analyze the influence of printing speed, layer thickness, printing temperature, and filling density on tensile properties. The optimal tensile performance of PEEK samples was observed at a printing speed of 60 mm/s, a layer thickness of 0.2 mm, a temperature of 370 °C, and a filling density of 40%. Wu et al. [29] investigated the influence of layer thickness and the raster angle on the mechanical properties of 3D-printed PEEK. The optimal mechanical properties of samples were found at a layer thickness of 0.3 mm and a raster angle of 0°. In a separate study, Dawoud et al. [30] studied the comparison between FDM and injection molding to investigate the effect of the processing method on the mechanical behavior of ABS and explored the optimal FDM parameters to enhance the mechanical properties.

From the above-mentioned published literature, it can be seen that most research focuses on the influence of FDM processing parameters on the mechanical properties of PEEK. However, PEEK parts prepared by FDM always have some defects generated during the processing. Consequently, it is essential to investigate the defects caused by the FDM processing of PEEK and analyze the impact of these defects on the mechanical properties. Moreover, considering the service environments of dynamic loads at different temperatures, the dynamic thermomechanical properties of PEEK deserve a detailed investigation.

In this study, a set of commonly used FDM processing parameters was selected. Tensile, compression, and bending tests were performed to evaluate the mechanical properties of FDM-prepared PEEK. The tensile failure mechanism of PEEK prepared by FDM and extrusion modeling (EM) was analyzed by fracture morphology observation. In order to analyze the differences between the tensile properties of FDM-prepared and EM-prepared PEEK, the crystallinity of PEEK prepared by FDM and EM was calculated by the results of the differential scanning calorimetry (DSC) test. Additionally, the dynamic mechanical analysis (DMA) for FDM-prepared PEEK was conducted to study the changes in dynamic thermomechanical properties of parts in certain service environments. The research conclusions have significance for improving the mechanical properties and service environment of FDM-prepared PEEK parts. The obtained results can also provide a reference for other polymer parts manufactured by FDM.

## 2. Materials and Methods

### 2.1. FDM Processing

A PEEK filament (PEEK 551G) with a diameter of 1.75 ± 0.05 mm was utilized to manufacture PEEK samples. The FDM equipment involved in this study was the ENGINEER-Q300, JUGAO-AM Co., Ltd, Weinan City, China. During the printing process, the nozzle diameter, printing speed, and filling gap were determined to be 0.4 mm, 40 mm/s, and 0.4 mm, respectively. Some of the literature showed that higher nozzle temperatures can reduce the viscosity of the molten PEEK, resulting in better rheological properties of the molten PEEK [31,32]. The good flowability and low viscosity of the extruded material improve the diffusion of newly extruded PEEK to the lower layer, resulting in strong interlayer adhesion and strength. In addition, a high nozzle temperature helps to achieve excellent adhesion between molten filaments. Additionally, a higher nozzle temperature can help to increase the crystallinity of PEEK, thus improving the mechanical properties of PEEK parts [33]. Therefore, during the printing process, a higher nozzle temperature of 420 °C was chosen.

Before printing, 3D modeling software was used to build the model according to the actual shape and size of the test sample. After the modeling was completed, the file was exported into the STL format recognized by the printer, and the STL file was imported into the slicing software of the printer. Subsequently, the thickness of each layer, the width of the path, the printing speed, the printing temperature, and other data information were set in the slicing software. Finally, the slicing software sliced the 3D model and generated the moving path information for each layer printed by the nozzle.

Once printing started, the thermoplastic filament was fed into the heating device by the friction force of the filament feeding mechanism and then melted under the high temperature of the heating device. Afterwards, the molten material flowed into the nozzle and was extruded on the printing platform through the nozzle. The molten material after extrusion was quickly cooled and solidified due to the stop of heating and contact with the low-temperature atmosphere. At the same time, the computer controlled the movement path of the nozzle. When the filament finished filling the physical contour of the first layer, the nozzle rose to a preset height, or the printing platform descended to the same height to fill the physical contour of the next layer. Hence, the filling material was bonded to the previous layer to deposit layer by layer until the printing of the entire target model was completed. To ensure uniform crystallization, the printed samples were kept warm for two hours at 300 °C after FDM processing.

### 2.2. Mechanical Tests

In practical applications, PEEK parts are subjected to various service conditions, so various mechanical tests, including tensile, compression, and bending tests, were conducted to test the mechanical properties of FDM-prepared PEEK.

#### 2.2.1. Tensile Test

**Figure 1:**
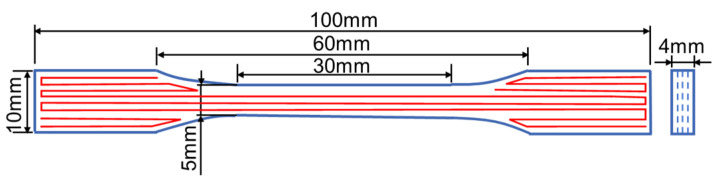
Configuration and dimension of tensile specimens.

The tensile properties of PEEK were investigated using an electronic universal testing machine (Z050, ZwickRoell Corporation, Ulm, Germany). The configuration and dimension of the specimens followed the 1BA type small specimens specified in GB/T 1040.2-2006 [34], with a gauge length of 25 mm, as shown in Figure 1. The loading direction was parallel to the infill line direction, with a test speed of 1 mm/min. In addition, some tensile samples manufactured by EM as a widely used processing method for PEEK were also prepared to compare the differences in mechanical properties under different processing methods. In each group, at least five samples were tested to ensure repeatability. The tensile properties of these two kinds of different specimens were analyzed based on three indicators: elastic modulus, yield strength, and fracture elongation.

#### 2.2.2. Compression Test

**Figure 2:**
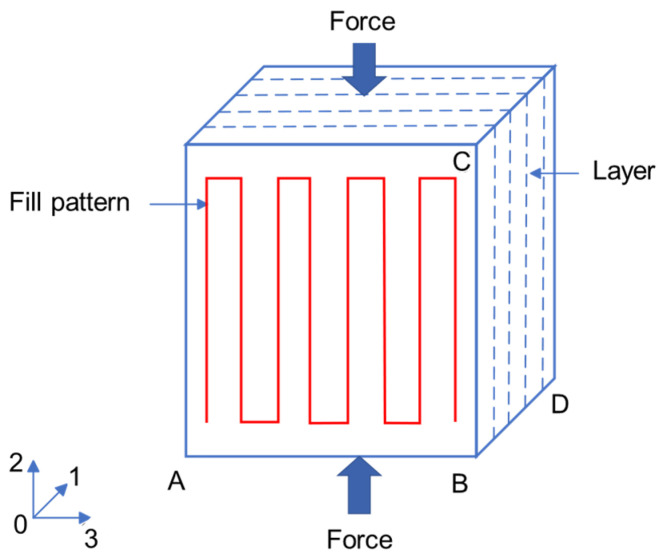
Schematic diagram of the compression test.

The compression properties of PEEK were investigated using an electronic universal testing machine (E45.105-B, MTS Systems Corporation, Minneapolis, MN, USA). The configuration and dimension of the specimens for the measurement of compressive modulus and strength followed the preferred sample size given in GB/T 1041-2008 [35]. The compression force direction was parallel to the infill line direction, with a test speed of 1 mm/min. The shape and fill pattern of the compression specimen are shown in Figure 2, in which the 203 plane was parallel to the workbench, and the 01 direction was the layer direction for additive manufacturing. For the measurement of compressive modulus, the lengths of AB, BC, and BD were set as 10 ± 0.2 mm, 50 ± 0.2 mm, and 4 ± 0.2 mm, respectively. For the measurement of compressive strength, the lengths of AB, BC, and BD were set as 10 ± 0.2 mm, 10 ± 0.2 mm, and 4 ± 0.2 mm, respectively. At least five samples were tested to ensure repeatability.

#### 2.2.3. Bending Test

**Figure 3:**
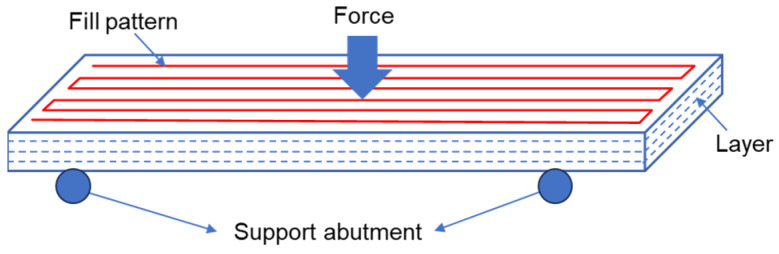
Schematic diagram of the bending test.

The bending test was conducted on the electronic universal material testing machine (Instron 5967, Instron Corporation, Boston, MA, USA). The dimensions of the test specimens followed the recommended sample size given in GB/T 9341-2008 [36]. The length, width, and height of the samples were set as 80 ± 2 mm, 10.0 ± 0.2 mm, and 4.0 ± 0.2 mm separately, with a span of 60 mm. The direction in which the specimen withstood the bending force was perpendicular to the infill line direction, with a test speed of 2 mm/min, as shown in Figure 3. At least five samples were tested to ensure repeatability.

### 2.3. Scanning Electron Microscope (SEM) Observation

The processing method has a great influence on the mechanical properties of the sample. A fracture morphology analysis of the tensile fracture through a scanning electron microscope (SEM) observation should be conducted to analyze the specific influence mechanism. The microscopic morphology of the fracture after the tensile test was observed by the JSM-IT300 electron microscope (SEM-JEOL, Tokyo Metropolis, Japan). Since PEEK is not conductive, the fracture section needed to be gold-sprayed before testing.

### 2.4. Differential Scanning Calorimetry (DSC) Test

FDM is a high-temperature processing method. Therefore, after the PEEK samples were manufactured by FDM processing, their crystallization condition may have changed. Crystallinity has a significant effect on the mechanical properties of polymer materials. Since differential scanning calorimetry (DSC) is a simple and accurate method to measure the crystallinity of polymer materials, the crystallinity was measured by this method to analyze the crystallization difference in the test samples.

FDM-prepared and EM-prepared PEEK samples were cut into 5.09 mg and 5.26 mg separately and dried at 80 °C for half an hour. The DSC test was carried out on the DSC8500 Differential Scanning Calorimeter (PE CO., Ltd., Waltham, MA, USA). The test temperature range was set at 80 °C to 400 °C, and the heating rate was 10 °C/min [37,38,39]. The obtained curve had a melting peak due to the existence of the crystalline region. If the melting enthalpy is known for the complete crystallization of crystalline PEEK, the crystallinity of PEEK can be calculated by the formula as follows [40]:

(1) $$W_{c}=\frac{\Delta H_{f}}{\Delta H_{f}^{*}}\times 100\%$$

where $W_{c}$ is the crystallinity of PEEK, $\Delta H_{f}$ is the melting enthalpy of the sample, and $\Delta H_{f}^{*}$ is the melting enthalpy when PEEK is completely crystallized. According to the existing literature [41], the melting enthalpy for completely crystalline PEEK is set as $\Delta H_{f}^{*}=130\;J/g$.

### 2.5. Dynamic Mechanical Analysis (DMA)

Because of the viscoelasticity of PEEK, it exhibits changes in mechanical properties when subjected to dynamic loads at different temperatures. The study of the changes in dynamic thermomechanical properties of parts with dynamic loads in certain service environments is of great significance for the correct use of materials and guidance for improving the service environment of parts. The dynamic thermomechanical properties of PEEK prepared by FDM were tested by the forced non-resonance method. The sample size was 20 mm × 6 mm × 2 mm. Since FDM is a kind of thermoforming processing, the printed sample should be left standing at room temperature for 48 h before the test to eliminate the residual stress in the sample.

The test was carried out on the DMA8000 dynamic mechanical analyzer (PE CO., Ltd., Waltham, MA, USA), which can perform multi-frequency scanning of the same sample in temperature scanning mode. The test temperatures ranged from 25 °C to 300 °C, and the heating rate was 2 °C/min. The storage modulus, loss modulus, and loss factor of the sample were measured under the conditions of 1 Hz, 5 Hz, 10 Hz, and 25 Hz.

## 3. Results and Discussion

### 3.1. Mechanical Property Analysis

**Figure 4:**
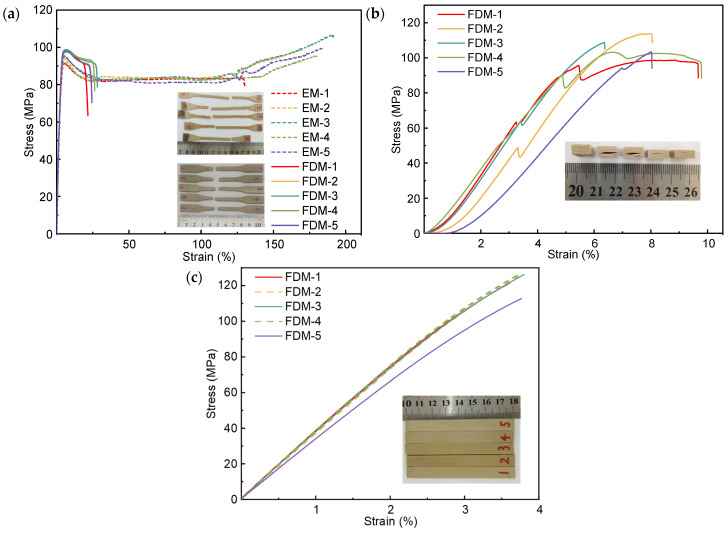
Stress–strain curves of mechanical tests for PEEK samples: (**a**) tensile test, (**b**) compression test, and (**c**) bending test.

The tensile, compression, and bending stress–strain curves of the specimens were obtained through mechanical tests, as shown in Figure 4. The insets in Figure 4 are the corresponding samples after tests.

As displayed in Figure 4a, the EM-prepared specimens exhibited large strain softening after the elastic stage. Specifically, a large strain occurred in the uniform extension stage with a small amount of change for tensile stress. Subsequently, the strain hardening appeared, and the stress rose until the sample was fractured. Differently, the FDM-prepared samples displayed necking after softening, and the mechanical property of the sample declined rapidly until fracture.

**Figure 5:**
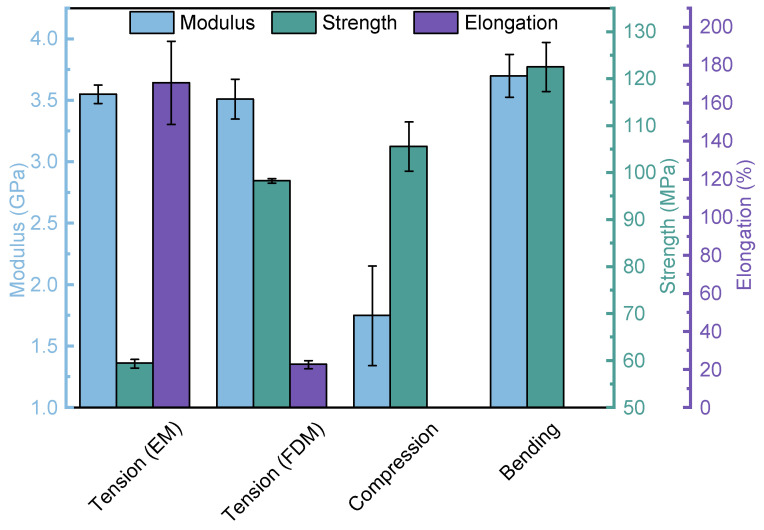
Mechanical properties of PEEK specimens.

The tensile properties of PEEK specimens prepared by FDM and EM are summarized in Figure 5. For FDM-prepared PEEK samples, the elastic modulus, yield strength, and elongation were 3.51 ± 0.16 GPa, 98.3 ± 0.49 MPa, and 22.86 ± 2.12%, respectively. Since the FDM processing parameters have an influence on the tensile properties of the PEEK parts, our results were compared with other published values, as displayed in Table 1. It can be seen that the yield strength value in this work is higher than others, and the values of elastic modulus and elongation are at the intermediate level among all the values. For EM-prepared PEEK samples, the corresponding results were 3.55 ± 0.07 GPa, 59.44 ± 0.93 MPa, and 170.78 ± 21.81%. It could be concluded that different processing methods could influence the mechanical properties of PEEK. Compared with EM processing, FDM processing could increase the yield strength of PEEK by 65.38%. Additionally, the elongation of PEEK formed by FDM processing was reduced by 86.61% compared to that formed by EM processing.

**Figure 6:**
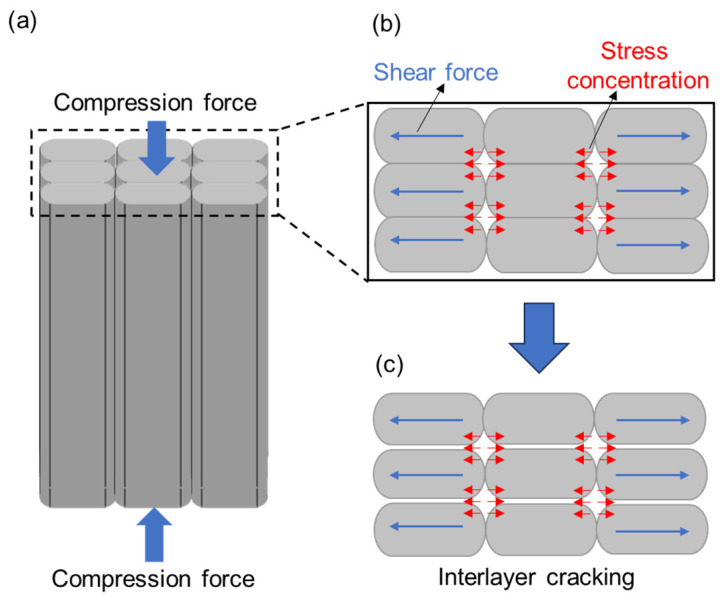
A schematic diagram of the compression failure mechanism for FDM-prepared PEEK: (**a**) the initial state of the sample in the compression test, (**b**) the enlarged top view of the sample, and (**c**) the formation of interlayer cracking.

As displayed in Figure 5, the obtained compressive modulus and strength were 1.75 Gpa and 105.6 Mpa, respectively. When the compression force was along the infill line direction, the samples tended to be damaged in the form of interlayer cracking, as shown in the inset of Figure 4b. The characteristic of FDM processing is that the product was formed by stacking each layer of material from bottom to top. The nozzle extruded high-temperature molten material to fill along a given path. In the process of FDM processing, due to the high surface tension of the molten filament, it was difficult to flow and expand but solidified quickly, thus leading to the formation of regularly distributed micropores between the molten filament overlap, as shown in Figure 6a. The shear force generated in each layer of the FDM-prepared PEEK caused stress concentration near micropores, which could easily lead to the enlargement of micropores and ultimately result in interlayer cracking. The schematic diagram of the formation of interlayer cracking is shown in Figure 6b,c.

**Figure 7:**
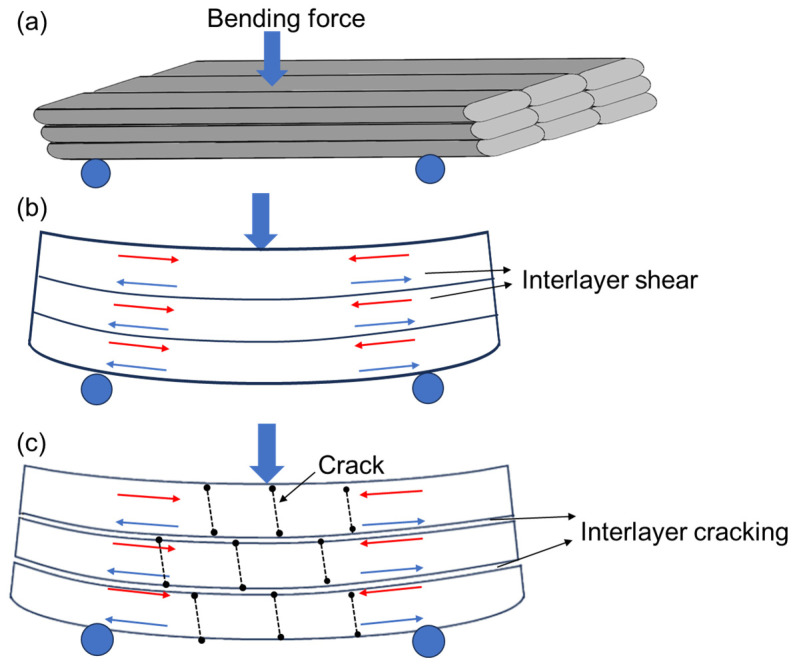
A schematic diagram of the interlayer shear in the bending test for FDM-prepared PEEK: (**a**) initial state of the sample in the bending test, (**b**) interlayer shear in the sample, and (**c**) interlayer cracking and internal crack formation after bearing the bending load.

The bending modulus and strength were measured as 3.7 Gpa and 122.49 Mpa, respectively, as shown in Figure 5. As displayed in Figure 7, due to the interlayer shear stress exceeding the interlayer shear strength, interlayer cracking occurred in the sample. In addition, when the bending stress exceeded the bending strength, cracks appeared inside FDM-prepared PEEK. However, the internal cracks were discontinuous and terminated at the interface of adjacent two layers because of the interlayer cracking, as shown in Figure 7c.

### 3.2. Tensile Failure Mechanism

**Figure 8:**
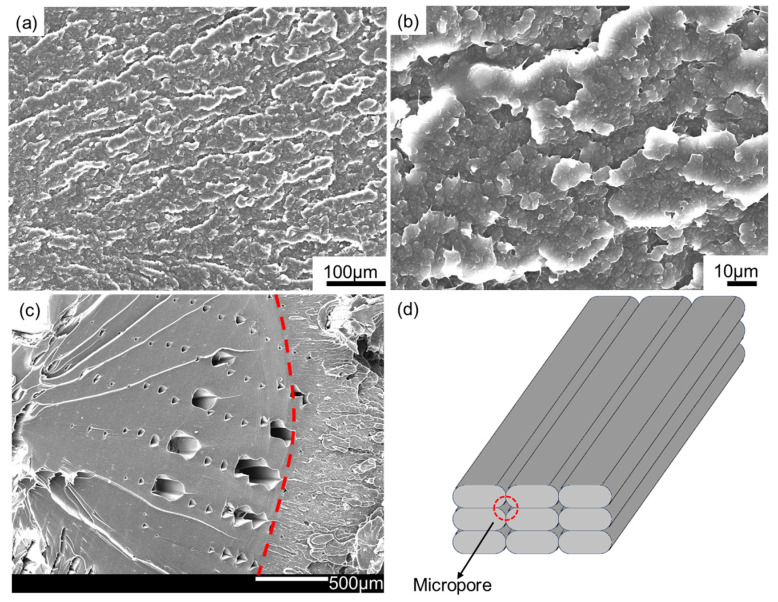
Fracture morphology of PEEK samples formed by different processing: (**a**,**b**) EM, (**c**) FDM, and (**d**) a schematic diagram of micropore formation during FDM processing.

By observing the microstructure of the fracture morphology, the tensile failure mechanism of PEEK prepared by different processing methods can be analyzed. The fracture morphology of PEEK samples after the tensile test is shown in Figure 8. It could be observed from Figure 8a,b that the crystal grains were sheared in the fractured EM-prepared sample, and there were crazes at the edge where the material was torn and destroyed. The fractured sample needed to absorb a large amount of energy due to the craze toughening mechanism and the formation of a rough fracture surface [44]. The material was seriously plastically deformed, and the damage was mainly shear yielding, so it showed strong toughness.

For the FDM-prepared sample, as displayed in Figure 8c, there were a large number of regularly arranged micropores inside. The density of the sample measured by the Archimedes drainage method was 97.7%, and the micropores were roughly arranged in parallel along a straight line. The reason for the formation of micropores inside the tensile samples is the same as the samples for compression and bending tests, as shown in Figure 8d. Part of the micropores were connected by the boundaries during the tensile deformation. In the FDM processing, due to the difficulty of the molten material flowing and its rapid solidifying, regular micropores formed inside, which was also observed in the fracture morphology of thermoplastic polymers such as PLA and ABS [19,29,45].

The left and right parts of the fracture (as indicated by both sides of the red dashed line in Figure 8c) in the FDM-prepared sample showed different fracture characteristics. The parallel arc-shaped steps in the upper left corner exhibited a typical brittle fracture morphology. The fracture area shown on the right side was very rough, and many grains were sheared. It was the same as the fracture of the EM-prepared sample, which was a typical ductile fracture characteristic. Based on the proportion of brittle areas and the severity of the decrease in the toughness of the sample, the FDM-prepared sample was mainly a brittle fracture, accompanied by some ductile fracture features. The micropores inside caused a strong stress concentration phenomenon, which made it easy to become a source of crack propagation. However, the presence of the right part in the fracture guaranteed that the material had a certain toughness.

### 3.3. Crystallinity Analysis

**Figure 9:**
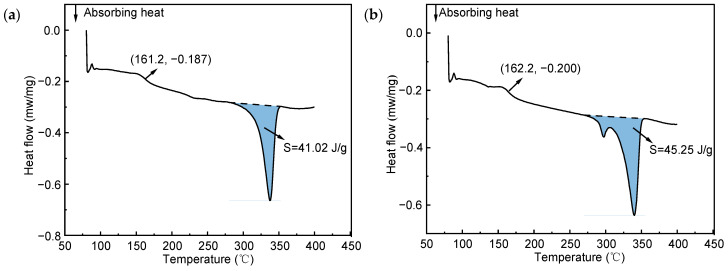
The DSC curves of samples prepared by different processing: (**a**) EM and (**b**) FDM.

The DSC curves of the EM-prepared and FDM-prepared samples recorded in the DSC experiment are shown in Figure 9. The glass transition temperatures (T_g_) of the EM-prepared and FDM-prepared samples were 161.2 °C and 162.2 °C, respectively, which were within the experimental error range. According to the melting peak area in the curves, the melting enthalpy of EM and FDM samples is calculated as $\Delta H_{f}=41.02\;J/g$ and $\Delta H_{f}=45.25\;J/g$, respectively.

The crystallinity of EM-prepared and FDM-prepared samples could be further calculated as 31.55% and 34.81%, respectively, according to Equation (1). Compared with the EM-prepared sample, the crystallinity of the FDM-prepared sample was increased by 10.33%. When PEEK is below T_g_, the molecular chains arrange in an orderly manner through crystallization. Thus, the mobility of the molecular chains is reduced and the intermolecular interaction is enhanced, and, thereby, the strength of the material increases and the toughness decreases. The crystallinity of PEEK samples prepared by FDM processing in this study is higher than that of FDM-prepared samples in the other literature [37,38]. Since the crystallinity of the material was higher, it could exhibit better mechanical strength.

It can be seen that the crystallinity of FDM samples is higher than that of EM samples, which is beneficial for increasing the yield strength of PEEK and reducing fracture toughness. Additionally, due to the directionality of the filament filling path along the tensile direction during the FDM manufacturing process of the sample, the polymer molecule chains will also maintain orientation in this direction, enhancing the yield strength of the material. This phenomenon is consistent with the research results of Tekinalp et al. [46]. Therefore, the FDM-prepared sample could show higher strength, even if there were a large number of regular micropores inside. However, the micropores will lead to rapid crack propagation and significantly reduce fracture elongation.

### 3.4. Dynamic Thermomechanical Analysis

**Figure 10:**
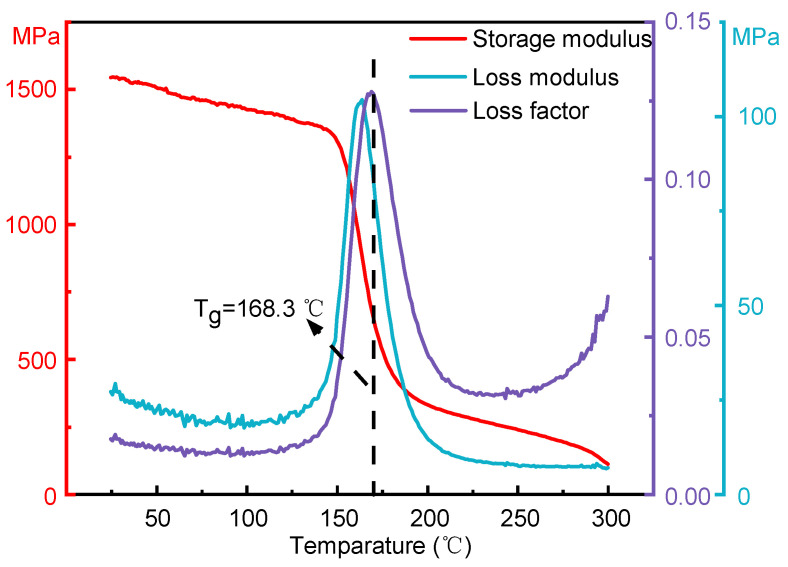
The curves of storage modulus, loss modulus, and loss factor of PEEK at a frequency of 1 Hz vs. temperature.

The dynamic thermomechanical properties of PEEK prepared by FDM processing under an alternating load of 1 Hz with variable temperatures are shown in Figure 10. When the temperature increased from 25 °C to 300 °C, the storage modulus of PEEK continuously decreased from 1545 MPa to 112 MPa, with a slower decrease in the high- and low-temperature regions. When the temperature was in the range of 148 °C to 180 °C, the sharp drop of storage modulus in the range indicated that PEEK had undergone a glass transition in this temperature range. Herein, the T_g_ of PEEK under the alternating load of 1 Hz was 168.3 °C. Within the glass transition zone, both the loss modulus and the loss factor rose rapidly at first and then dropped sharply. The peak value of the loss modulus was 104 MPa and that of the loss factor was 0.128. In the temperature range below 148 °C and above 225 °C, the loss modulus and loss factor were at a low level, which indicated that the loss factor in the material began to increase first and then decreased rapidly from 148 °C to 225 °C. In addition, it could be observed that the loss factor of PEEK increased with the increase in temperature after exceeding 250 °C, indicating that the material had entered the viscous flow transition zone.

**Figure 11:**
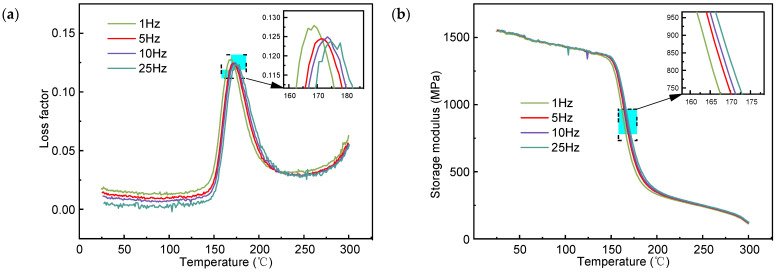
The curves of PEEK under different frequencies vs. temperature: (**a**) loss factor and (**b**) storage modulus.

The loss factor and storage modulus of PEEK at different frequencies as a function of temperature are shown in Figure 11. The T_g_ of PEEK indicated by the loss factor and storage modulus shifted towards the high-frequency direction of external forces. As shown in Figure 11a, below the T_g_ of PEEK, the loss factor under high-frequency external forces was lower than that under low-frequency external forces at the same temperature. At a temperature higher than the T_g_ of PEEK, the loss factor under high-frequency external forces was higher than that under low-frequency external forces at the same temperature.

The polymer is in the glassy state before the glass transition. From the perspective of the molecular chain segment motion, the change rate of external load in the high-frequency region is fast, while the motion response of the molecular chain is slow, which could be regarded as movement freeze. Moreover, the higher the frequency of the external load, the lower the internal friction loss. After the material has undergone a glass transition, it is in a highly elastic state, and the molecular chain segment is in active motion. When applied with high-frequency external loading, the motion is intensified, and the internal friction loss is increased. Moreover, the higher the external loading frequency, the greater the internal friction loss. As indicated in Figure 11b, the external loading frequency had little influence on the storage modulus under low temperatures (below 148 °C) and high temperatures (above 180 °C). Within the glass transition zone, the storage modulus of PEEK under high-frequency external forces was greater than that under low-frequency external forces at the same temperature.

PEEK is often used in manufacturing structural components due to its excellent mechanical properties. From Figure 10, it can be seen that when PEEK is in the glassy state, its storage modulus is the highest, indicating excellent stiffness and strength. Consequently, the service environment of PEEK needs to be within this temperature range. In addition, as the T_g_ of PEEK increases with the rise of external force frequency, appropriately increasing the external load frequency to improve the T_g_ can expand the temperature range for the use of PEEK components. Additionally, when PEEK is in the glassy state, the higher the frequency of the external load, the lower the internal friction loss. Therefore, increasing the external load frequency is beneficial to enhance the mechanical properties of PEEK.

**Figure 12:**
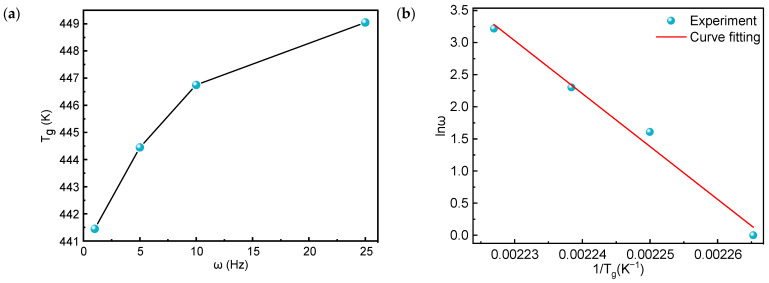
Experimental results of T_g_ and curve fitting between lnω and ${1/T}_{g}$: (**a**) T_g_ of PEEK at different frequencies and (**b**) fitting curve between lnω and ${1/T}_{g}$.

The total energy required for structural reorganization or the motion of materials is called activation energy [47]. For polymers, the activation energy of glass transition is a significant parameter since this value determines the ease of glass transition. The lower the activation energy of the glass transition, the less energy the material absorbs for the glass transition. Li et al. [48] demonstrated that the activation energy based on the peak of the loss factor is more reliable than the activation energy based on the peak of the loss modulus. Consequently, by determining the temperature corresponding to the peak of each loss factor, the T_g_ of PEEK at different frequencies is indicated in Figure 12a. According to the principle of time–temperature superposition, the loss factor peaks determined at different test frequencies can be superposed. When the temperature range does not change significantly, it is suitable to use the Arrhenius equation to determine the activation energy of chemical reactions. According to the Arrhenius equation, the temperature dependence of the test frequency can be expressed as the following [48]:

(2) $$\omega =\omega_{0}\exp(-\frac{\Delta E}{RT})$$

where ω denotes frequency, $\omega_{0}$ is the pre-exponential factor, ΔE is the activation energy (J/mol), R is the gas constant (8.314 J/mol·K), and *T* is the thermodynamic temperature in unit K.

Due to the variation of test frequencies, the glass transition temperatures change accordingly. The relationship can be expressed as follows:

(3) $$\frac{\omega_{1}}{\omega_{2}}=\frac{\exp(-\frac{\Delta E}{RT_{g1}})}{\exp(-\frac{\Delta E}{RT_{g2}})}$$

By taking the logarithm of both sides of this equation, Equation (4) can be obtained.

(4) $$\ln(\frac{\omega_{1}}{\omega_{2}})=\frac{\Delta E}{R}(\frac{1}{T_{g2}}-\frac{1}{T_{g1}})$$

Therefore, the temperature-dependent activation energy of the glass transition can be obtained as follows:

(5) $$\Delta E=-R\frac{d(\ln\omega )}{d(\frac{1}{T_{g}})}$$

According to the data in Figure 12a, the relationship between the $T_{g}$ of PEEK and lnω was obtained as shown in Figure 12b. The slope of the fitted curve is −8239.99. Combining the obtained value and the gas constant R, the activation energy of the glass transition was obtained as ΔE = 685.07 kJ/mol. This value is higher than the glass transition activation energy of other special engineering plastics, such as polyimide [49] and a liquid crystalline polymer [50], indicating that PEEK has a high energy barrier for molecular chain movement during glass transition, which helps maintain good mechanical properties under high-temperature conditions.

## 4. Conclusions

In this work, the mechanical properties of PEEK based on FDM are experimentally investigated, and the failure mechanisms are evaluated. The dynamic thermomechanical tests under different frequencies provide useful guidance for the usage of PEEK components. The main conclusions could be drawn as follows:

1. The micropores inside FDM-prepared PEEK will cause a strong stress concentration, leading to a significant fracture elongation decrease. Compared with PEEK manufactured by EM, the yield strength of PEEK manufactured by FDM was increased by 65.38%. The primary reason is probably that the crystallinity of FDM-prepared PEEK is 10.33% higher than that of EM-prepared PEEK. Another reason is the directionality of the filament filling path along the tensile direction during the FDM manufacturing process of the sample, which enhances the yield strength of the material;
2. When subjected to compression loads, the shear force generated in each layer of the FDM-prepared PEEK caused stress concentration near the micropores formed in the process of FDM, which resulted in interlayer cracking. When subjected to bending loads, due to the interlayer shear stress exceeding the interlayer shear strength, interlayer cracking occurred in FDM-prepared PEEK. Additionally, when the bending stress exceeded the bending strength, discontinuous internal cracks would appear.
3. Based on the Arrhenius equation, the glass transition activation energy of PEEK was obtained as ΔE = 685.07 kJ/mol. This value is higher than the glass transition activation energy of other special engineering plastics, such as polyimide and a liquid crystalline polymer, which helps maintain good mechanical properties under high-temperature conditions.

PEEK has become a promising material in aerospace engineering due to its excellent comprehensive properties and the advantages of light weight. In addition, due to the biocompatibility of PEEK, it also has significant research significance in biomedical applications. FDM, as a simple and mature additive manufacturing technology, is widely used to process complex-shaped components and helps improve production efficiency. Consequently, PEEK parts manufactured by FDM processing have potential applications in different fields. One possible application in aerospace engineering is aircraft load-bearing brackets, which use thermoplastic polymers instead of metal materials. Additionally, early clinical trials of PEEK for cervical and lumbar fusion have been initiated, which is expected to be put into practical medical applications. The research conclusions in this paper have significance for improving the mechanical properties and service environment of PEEK parts manufactured by FDM processing.

## Figures and Tables

**Table 1:** Summary of the tensile properties of different FDM-prepared PEEK.

PEEK	Yield Strength (MPa)	Elastic Modulus (GPa)	Elongation (%)	Processing Parameters	Reference
PEEK-1000 bar (Zhongshan Yousheng Plastic Materials Co., Ltd.)	40	0.52	14.3	Nozzle temperature 370 °C, printing speed 60 mm/s, layer height 0.2 mm, and 40% infill	[23]
Victrex^®^ PEEK 450G	82.58	3.80	110.97	Nozzle temperature 410 °C, bed temperature 100 °C, 100% infill, and 0.1 mm layer height	[42]
KetaSpire^®^ KT-880 high flow PEEK	66.2	3.15	3.10	Nozzle temperature 390 °C, bed temperature 100 °C, printing speed 1000 mm/min, extrusion width 0.48 mm, and 100% infill	[43]
VESTAKEEP 3300G	96.9	3.54	4.57	Nozzle temperature 300 °C, bed temperature 100 °C, ambient temperature 30 °C, printing speed 30 mm/s, and 0.15 mm layer height	[32]
Thermax™ PEEK Natural, 3DXTECH	87.53	3.031	23.454	Nozzle temperature 410 °C, bed temperature 130 °C, chamber temperature 90 °C, layer height 0.2 mm, and printing speed 50 mm/s	[31]
PEEK 551G	98.3	3.51	22.86	Nozzle diameter 0.4 mm, printing speed 40 mm/s, nozzle temperature 420 °C, and filling gap 0.4 mm	This work

## Data Availability

Data are contained within this article.
